# Managing Nutrition Impact Symptoms in Cancer Cachexia: A Case Series and Mini Review

**DOI:** 10.3389/fnut.2022.831934

**Published:** 2022-03-03

**Authors:** Adam Khorasanchi, Srinidhi Nemani, Sudeep Pandey, Egidio Del Fabbro

**Affiliations:** ^1^Department of Internal Medicine, Division of Hematology, Oncology, and Palliative Care, Virginia Commonwealth University, Richmond, VA, United States; ^2^Virginia Commonwealth University, Richmond, VA, United States

**Keywords:** nutrition impact symptoms, malnutrition, cancer, cachexia, review

## Abstract

Malnutrition is common in cancer patients and can occur throughout a patient’s disease course. The contributors to the clinical syndrome of cancer cachexia are often multifactorial, and produced by the cancer and associated pro-inflammatory response. Since cancer cachexia is a multifactorial syndrome, a multimodal therapeutic approach is ideal. A key component of therapy is identifying and managing symptom barriers to adequate oral intake, known as nutritional impact symptoms (NIS). NIS are associated with reduced intake and weight loss in patients with advanced cancer, and aggregate NIS are a predictor of survival in patients with Head and Neck Cancer and in patients undergoing surgery for esophageal cancer. Currently, there are no guidelines regarding the specific management of NIS in oncology patients. Experience from specialist centers suggest relatively simple assessments and inexpensive interventions are available for the diagnosis and treatment of NIS. We present three patient cases from a cachexia clinic, where NIS management decreased symptom burden and improved clinical outcomes such as weight and physical performance.

## Introduction

Weight loss is considered the key criterion for cachexia and malnutrition (1). Since both of these conditions are defined by the presence of weight loss (2), the notion of managing nutrition impact symptoms (NIS) is an essential component of multimodal, multidisciplinary treatment. A working definition of NIS are symptoms that compromise food intake and in turn drive body weight loss (3). Symptoms, complications of cancer, anticancer treatment, or medical co-morbidities can interfere with a patient’s appetite and ability to eat or digest food (4). Barriers to adequate oral nutritional intake may include symptoms such as nausea, constipation, depression, pain, and vomiting (5).

Prompt recognition and treatment of NIS is critical since inexpensive medications and supportive care measures are effective for the majority of these symptoms. The identification and management of NIS may also have implications for the design of clinical trials involving pharmacological interventions for cancer-related cachexia or malnutrition. For example, a patient in a clinical trial with inadequately managed nausea or oral pain may not experience the beneficial effects of an investigational drug for cancer cachexia.

There are several recently published clinical guidelines for managing cancer cachexia and weight loss. All refer to NIS, either directly or indirectly as an important component of clinical care. However, the specifics and priority of NIS management varies among the guidelines, and managing NIS are never foremost among the recommendations. American Society of Clinical Oncology (ASCO) management guidelines note that “uncontrolled NIS” are frequently encountered in patients with cachexia and are associated with adverse outcomes such as weight loss and decreased survival (6). European Society of Medical Oncology (ESMO) guidelines (7) recommend that at risk patients be assessed for NIS, GI dysfunction, chronic pain and psychosocial distress. European Society for Parenteral and Enteral Nutrition (ESPEN) guideline charts for practical clinical use (8), recommend objective and quantitative assessment of NIS. They note that addressing NIS may improve quality of life (9, 10) in oncology patients. Clinical Oncology Society of Australia (COSA) guidelines (11) emphasize a multidisciplinary approach and note that alongside medical nutrition therapy, interventions should include management of treatment side effects and symptoms. Regarding dietary counseling and exercise, although the evidence is limited, counseling by a dietician is recommended by all the guidelines, and some include specific recommendations for protein (e.g., at least 1.2 g/kg/day) and calorie intake (at least 25–30 kcal/kg BW/day) (8). COSA, ESPEN, and ESMO recommend individualized prescriptions that include resistance exercise in addition to aerobic exercise for maintaining muscle strength and mass. Based on systematic review the ASCO guidelines concluded that at present, evidence remains insufficient for a recommending exercise in cachexia patients. Our review paper provides an overview of the existing literature on NIS in oncology patients; and we offer specific recommendations regarding the pharmacological management of NIS. A case series of three patients with NIS who presented to our cachexia clinic, are included, illustrating our management of NIS and their potential for improving outcomes. However, it is important to note that in addition to NIS, other *non-symptom* barriers such as poor communication, conflicting advice and financial constraints may impede the management of cachexia (12).

## Methods

For the mini-review of NIS, we used the search term “Nutritional Impact Symptoms” in PubMed. The search revealed 147 papers in between 1997 and 2022. Only articles in English were included and studies focused exclusively on pre-clinical aspects of cachexia were excluded. The greatest number of studies were published in 2019 (13).

The clinical cases were selected based on their ability to return for follow up visits. Capturing their baseline assessments and at least one follow-up visit was required. Unfortunately, the majority of patients had only a single baseline visit and were unable to return for a variety of reasons including disease progression, complications requiring hospital admission or increased burden of outpatient visits.

## Clinical Cases

The following cases illustrate the potential benefit of identifying and managing NIS in oncology patients with weight loss. All three patients experienced improved symptom burden and physical performance. We should emphasize that these three patients did not receive specific medications known to be effective for cancer-related anorexia, such as megestrol acetate or corticosteroids. Based on their BMI and percentage weight loss all three patients are classified as grade 4 in the grading system developed by Martin et al. (14). All the patients attended a specialist cachexia clinic staffed by a multidisciplinary team including palliative care physician, dietitian, physical therapist, and social worker or psychologist.

### Case 1

**FIGURE 1:**
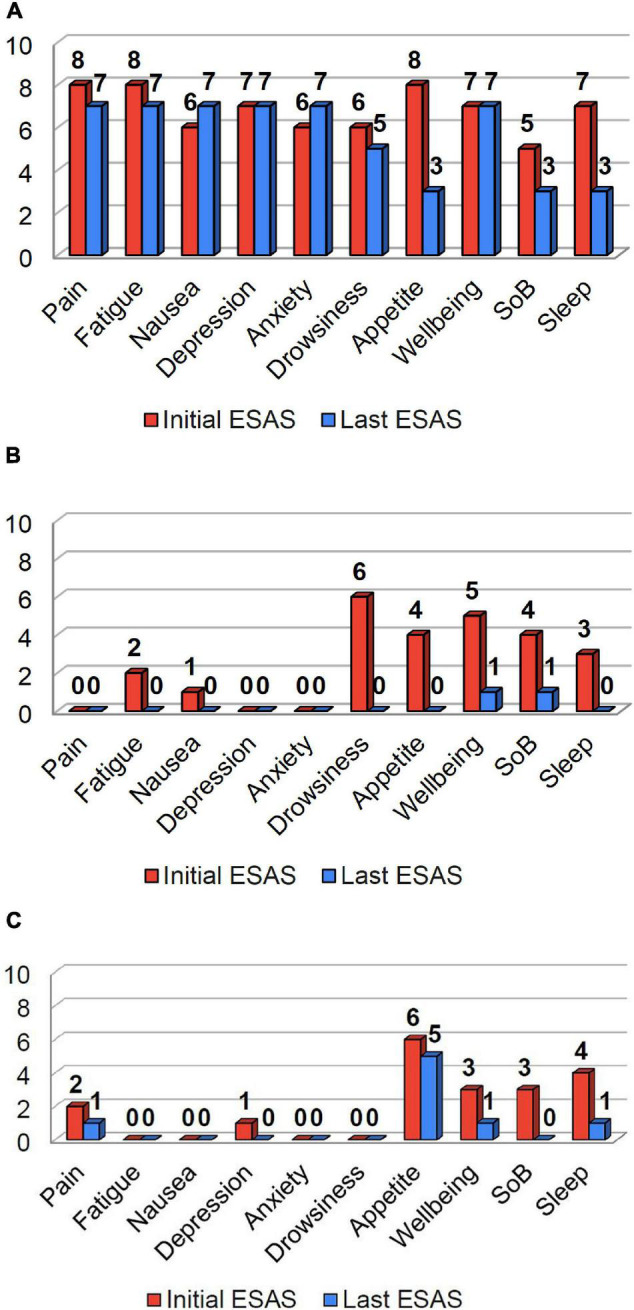
Initial (red) and last ESAS scores (blue) for patients in cases 1-3 are shown above. The patient in Case 1 **(A)**, reported pain, fatigue, and loss of appetite as their most significant symptoms. The patient in Case 2 **(B)** reported drowsiness, loss of appetite, well-being, and shortness of breath as their most significant symptoms. The patient in Case 3 **(C)** reported loss of appetite, wellbeing, shortness of breath, and poor sleep as their most significant symptoms. Following treatment, there was a substantial decrease in symptom burden for all three patients **(A–C)**, with improvements in a majority of their individual and total ESAS scores.

A 55-year-old male with recurrent stage IVA squamous cell carcinoma of the tongue base, no longer on anticancer treatment, but previously treated with cisplatin and radiotherapy, presented for management of pain and cachexia. The patient had a body mass index (BMI) of 15.4 kg/m^2^, and experienced a 17% weight loss over the past 3 months. Pain, fatigue, and loss of appetite were the most severe symptoms according to the initial Edmonton Symptom Assessment Scale (ESAS) (Figure 1A). Physical performance measures were notable for short physical performance battery (SPPB) score 6/12, handgrip strength 33 kg, and 6 min walk (6MW) score 485 ft. Methadone and duloxetine were prescribed for pain, metoclopramide for nausea and early satiety, and testosterone replacement for hypogonadal symptoms (total testosterone level 105 ng/dL). He received psychosocial support from the palliative care physician in the form of expressive supportive counseling, and brief motivational interviewing. He was referred to the supportive care psychologist for cognitive behavioral therapy. Eight weeks following treatment, he achieved an 11% weight gain (5 kg), increased BMI (17.3 kg/m^2^), improved SPPB (9/12), handgrip strength (38 kg), and 6MW (1,252 ft.). Symptom burden decreased, with improvements in more than half of the individual ESAS scores and total ESAS score decreased from 68 to 56 (Figure 1A). An individualized exercise program was recommended by a physical therapist, which included walking three times per week along with strength exercise using resistance bands. He declined referral to a dietician, and received nutritional advice from the nurse and physician in the clinic.

### Case 2

A 57-year-old female patient with colorectal carcinoma and metastases to the liver, spleen, and peritoneum, presented for management of weight loss. Prior treatments included folinic acid (leucovorin), fluorouracil (5-FU), and oxaliplatin (FOLFOX), and radiotherapy to the spleen. BMI was 22.5 kg/m^2^, and she reported 22% weight loss over the past six months. Drowsiness, loss of appetite, well-being, and shortness of breath were the most significant symptoms according to the initial ESAS (Fig. 1B). Physical performance measures were notable for a SPPB score 11/12, and 6MW score 1,180 ft. She was treated for Vitamin D deficiency; underwent a home exercise program that included resistance bands and aerobic activity; given laxatives for opioid induced constipation; and received nutritional counseling. In addition, she received radiotherapy to reduce the tumor burden of her abdominal metastases. Six weeks following treatment, she experienced an 11% weight gain (8 kg), increased BMI (24.3 kg/m^2^), and improvement of SPPB (12/12) and 6MW (1,388 ft.). There were improvements across all individual symptoms and total ESAS score decreased from 25 to 2 (Figure 1B). Other than expressive supportive counseling she required no additional psychosocial support.

### Case 3

A 70-year-old female with metastatic neuroendocrine small cell lung cancer presented with weight loss and poor appetite. Previously, she received two lines of therapy, and recently started ipilimumab and nivolumab. Evaluation revealed a BMI of 18.25 kg/m^2^ and 20% weight loss. Loss of appetite was the most severe symptom according to the initial ESAS (Figure 1C). Physical performance measures were notable for SPPB score 7/12, handgrip strength 43 kg and 6MW score 830 ft. She received mirtazapine for a combination of symptoms that included insomnia, poor appetite, and mild depression; a physical therapist prescribed an individualized exercise program; she received nutritional counseling; and psychosocial support in the form of expressive supportive counseling by the palliative care physician. Three weeks following treatment, she experienced a 2.7% weight gain (1.3 kg), increased BMI (18.6 kg/m^2^), SPPB score (9/12), handgrip strength 40 kg), and 6MW (1,060 ft.). There was also a decrease in symptom burden, with improvements across most individual symptoms and total ESAS score improved from 19 to 8 (Figure 1C).

## Review

### NIS Background

Disease-related malnutrition occurs frequently in oncology patients with the prevalence ranging from 40 to 80% (3). Patients with pancreatic, gastrointestinal, or head-and-neck cancer (HNC) are at higher risk for malnutrition while breast cancer patients have comparatively lower risk. NIS are common in oncology patients and are associated with decreased quality of life (QOL) and performance status. A prospective study of medical oncology clinic patients noted a symptom prevalence of 79 and 72% at 1 and 6 months after starting chemotherapy (10). Additionally, a retrospective study of 151 patients with solid tumors referred to a specialized cachexia clinic found a median of three NIS, and five or more NIS in 15% of patients (15).

### NIS Assessment Tools

Several validated tools can be used to assess NIS. The ESAS (16) and Patient-Generated Subjective Global Assessment (PG-SGA) are patient reported questionnaires used for symptom assessment and nutrition status respectively, in patients with cancer (17). Given that each questionnaire has advantages and some disadvantages, a combination of both tools has been used in a cachexia clinic to provide a more comprehensive assessment of patients’ NIS. The 10 item ESAS has the advantage of being able to assess individual symptom severity with a numerical rating scale including appetite, nausea, depression, anxiety, fatigue, shortness of breath, and drowsiness but does not include other pertinent NIS such as constipation, oral ulcers, early satiety, altered smell, or dysgeusia (18). These NIS are included in the PG-SGA, an American Dietetic Society-endorsed questionnaire identifying factors contributing to poor oral intake including symptoms that are consistent with upper gastrointestinal obstruction (dysphagia) or gastroparesis (feel full quickly). While the PG-SGA is able to identify several additional NIS (19), the binary answers (yes/no) do not allow for assessment of individual symptom severity. A validated abridged version, the PG-SGA Short Form, completed by patients in less than 5 minutes, allows for quick identification and prioritization of malnutrition in cancer patients. It is important to note that the short form of the PG-SGA has demonstrated value in prognostication, of several patient populations with cancer including elderly oncology patients (20); ambulatory patients receiving chemotherapy; patients with cervical cancer (21); and palliative care patients (22). Additionally, the Head and Neck patient symptom checklist (HNSC) is a comprehensive NIS assessment tool evaluating total symptom burden in patients with HNC. It includes fifteen symptoms in common with the PG-SGA; pain, anxiety, depression, nausea, vomiting, dry mouth, difficulty swallowing, altered smell, taste changes, sore mouth, lack of energy, feeling full, diarrhea, constipation, and anorexia, plus two additional symptoms (difficulty chewing and thick saliva). Patients rate the severity of each symptom and the degree to which it interferes with their dietary intake (23). For daily clinical practice, the ESAS plus the PG-SGA Short Form maybe the most pragmatic tools since both are widely used by palliative care clinicians and dieticians in daily practice. For example, Ontario’s cancer system has implemented the ESAS throughout the province in order to improve oncology care (24). Although the PG-SGA is the preferred nutrition-specific assessment tool, it was omitted in our series of three patients; however, it has since been implemented consistently into the clinic.

### Clinical Significance of NIS

Early recognition of NIS is critical given uncontrolled individual symptoms and/or aggregate symptom burden can compromise food intake and in turn drive body weight loss. Poor nutritional intake is linked to an increased risk of complications, poor response and tolerance to treatment, decreased survival, poor QOL (25), and increased length of hospital stay (26) and overall health care costs (27). Several preliminary studies have found an association between NIS burden and decreased survival. In a cohort of HNC patients, aggregate symptom burden was a significant independent predictor of reduced intake, weight loss, and survival, and equally significant to tumor stage and performance status in predicting impaired food intake. Loss of appetite, difficulty chewing, thick saliva, and pain were the NIS found to be significantly associated with reduced dietary intake (24). Similarly, studies in cancer patients undergoing surgery show greater NIS were associated with worsened global QOL (26), social and physical function at 6 months after surgery for esophageal cancer, regardless of pre-operative BMI or post-operative weight loss. More symptoms were associated with poorer survival in those with high pre-operative BMI and major post-operative weight loss. In another study of 135 surgical patients with cancer (5), three or more NIS were reported by 52% of patients, and PG-SGA symptoms found to increase the risk of malnutrition included anorexia, constipation, strange taste, mouth sores and others (depression, dental or financial problems). A multi-center study of 200 patients undergoing esophageal, gastric and pancreatic surgery, found malnutrition in 42 and 55% reported at least one NIS preoperatively. Vomiting was independently associated with the presence of malnutrition (13). In addition to specific cancer types [e.g., HNC (28)], age groups such as the elderly, appear to be at higher risk for malnutrition and a greater number of NIS than their younger counterparts (29). There is also strong evidence for an association between single symptom severity (pain and poor appetite) and decreased survival among oncology patients enrolled in anti-neoplastic treatment trials. A systematic review (30) of 30 EORTC trials found that more severe anorexia or pain scores were associated with decreased survival. This association may be an epiphenomenon, with severe symptoms being associated with disease that is more extensive. Nevertheless, the systematic review found that these symptom scores provide independent prognostic information, beyond that of the usual clinical and tumor characteristics.

### Physical Performance Measures

Objective measures of physical performance include the SPPB, 6MW, and handgrip strength test. We use all three measures for our cachexia clinic patients; however, the 6MW is most challenging from a time and “sufficient space” perspective. The SPPB is a valid, reliable and feasible measure of physical performance in older people, and there is increasing evidence for its prognostic value in oncology. Low SPPB scores are associated with higher rates of chemotherapy completion, fewer adverse events and hospitalizations in patients with Non-Small Cell Lung Cancer (31). Additionally, poor SPPB scores (SPPB < 9) were found to be associated with worse overall survival in elderly patients undergoing induction therapy for acute myeloid leukemia (AML) (32). The 6MW test measures endurance, cardio-respiratory fitness and was found to be significantly associated with mortality risk in a meta-analysis of 26 oncology studies (33). Handgrip strength using a handheld dynamometer is a fairly simple, rapid measure, and also shown to be associated with mortality risk in oncology; however, recent studies of investigational anti-cachexia agents have demonstrated mixed results with respect to its usefulness as an outcome measure. In the ACT-ONE trial patients with stage III/IV non-small cell lung cancer or colorectal cancer showed significant weight loss reversal, improved fat free mass, and maintained fat mass, along with improved handgrip strength (34). Conversely, in a study, involving advanced lung cancer patients there was a significant increase in several outcomes including lean body mass, yet the drug did not improve handgrip strength (35). In our case series, treatment of NIS resulted in improved SPPB and 6MW scores in all three patients. However, our patient in Case 3 did not experience improvement in handgrip strength.

### Non-pharmacologic Management of NIS

The role of symptom manaegment and supportive care is fundamental in addressing the many contributors to decreased oral intake. Moreover, a multidisciplinary team is required to provide superior quality supportive care (36). For instance, patients with dysphagia may benefit from nutritional interventions that focus on increasing the acceptability of complex food textures, thereby improving patient-reported swallowing outcomes. Preventative swallowing exercises taught by speech language pathologists may also be beneficial in reducing chemoradiotherapy-induced swallowing disorders (37).

Dietary counseling, which includes increasing energy dense foods (Goal: 25–30 kcal/kg/day, 1.2–1.5g protein/kg/day) (3), meal frequency, and the use of oral liquid nutritional supplements, represents another potentially effective therapy to manage weight loss. Dietary counseling can improve energy intake, body weight, and overall QOL in HNC patients receiving radiotherapy (38, 39).

Psychosocial support may prove to be an especially effective component of multimodality therapy for NIS. Although underused in the context of cachexia/nutrition clinics, psychosocial support has the potential to relieve distress and family conflict, support self-efficacy, decrease social isolation, improve body image, and adherence to treatment. For instance, a small study in the United Kingdom has demonstrated the feasibility of a nurse-delivered psychosocial intervention to mitigate weight and eating-related distress in patients with advanced cancer and their caregivers (40). Similarly, a preliminary, nurse-led educational intervention program conducted in China showed improvement of nutrition impact symptom clusters in patients with nasopharyngeal cancer (41).

Finally, polypharmacy is a common issue among older patients and under-appreciated as a contributor to symptoms in patients with cancer. Many drugs have the potential to affect nutritional status (42) by altering the sensory perception of taste, intestinal absorption, and metabolism or inducing the elimination of essential vitamins and minerals. In a prospective observational study of geriatric oncology patients, the prevalence of polypharmacy and excessive polypharmacy was found to be 45.2 and 8.6%, respectively, and was associated with poorer overall survival (43). Recognizing polypharmacy and de-prescribing specific drugs that are inappropriate for managing symptoms (e.g., prochlorperazine, promethazine, scopolamine for non-CINV) may be an especially effective, yet simple strategy for improving NIS (44).

### Pharmacologic Management of NIS

Pharmacologic treatment, using readily available inexpensive medications, remains a cornerstone of managing NIS. For example, patients with advanced cancer often have gastroparesis and dysmotility. These symptoms may be managed with dietary modification by eating small, frequent meals, and a prokinetic agent such as metoclopramide, enabling the stomach to accommodate more food and improving motility (Table 1). Although rare, tardive dyskinesia is an irreversible drug side effect, so the benefits of treatment beyond 3 months should be considered carefully since the duration and total cumulative dose of metoclopramide increase the risk of tardive dyskinesia.

**TABLE 1:** Pharmacologic management of NIS.

Nutrition impact symptoms	Pharmacological interventions
Early satiety; bloating; GERD	Metoclopramide 10 mg qid to q4h PO
Constipation	Laxatives, e.g., polyethylene glycol and senna
Nausea/vomiting	Metoclopramide 10mg qid or q4h po for non-CINV
	Olanzapine 10 mg qhs, particularly if CINV, and or depression are also present
	Mirtazapine 15 mg qhs if depression, insomnia, and anxiety are also present
Depressed mood or anxiety	Mirtazapine first choice
	Duloxetine, if neuropathic pain
Fatigue	Consider Testosterone replacement, Vitamin D
Dysgeusia	Zinc supplement trial for 2 weeks
Severe pain, for example mucositis	Opioids

*CINV, Chemotherapy-induced nausea and vomiting; GERD, gastroesophageal reflux disease.*

Nausea and vomiting are common in advanced cancer patients. The cause may be treatment related (chemotherapy, radiation, and surgery) or chronic, non-treatment related. In a randomized double blind phase 3 trial, olanzapine proved to be an effective agent for chemotherapy-induced nausea and vomiting (CINV) (45). In addition, olanzapine may be effective for other NIS, including depression and non-CINV (46), although Metoclopramide is still the preferred drug for non-CINV (but requires adequate dosing, 10mg qid or q4h po) (47). Constipation is exacerbated by medications such as opioids, gabapentin and ondansetron and is effectively managed with laxatives such as polyethylene glycol and senna, although few published trials have compared bowel regimens. Untreated, constipation can produce abdominal pain, nausea, and contribute to early satiety Therapies given to stimulate appetite include dexamethasone and progestational agents such as megestrol. While a short course of corticosteroids is effective, there are no long-term studies providing any guidance, while the concerns about increased thromboembolism and adverse effects on the endocrine system (hypoadrenalism and hypogonadism) diminish our enthusiasm for megestrol (48). Of note, neither of these medications were used in our case series and further discussion of specific agents for anorexia in cancer are beyond the scope of this review. Finally, other metabolic abnormalities such as hypogonadism, vitamin B12 deficiency, hypothyroidism, and hypoadrenalism may also contribute to anorexia and muscle wasting. Low testosterone in males and vitamin D deficiency (49) are commonly diagnosed (50), in patients with advanced cancer and associated with multiple symptoms including fatigue, muscle weakness and depressed mood. Replacement therapy should be considered in symptomatic patients with low serum levels (51).

Depressed mood is common in oncology patients. In those who are malnourished, a cross-sectional hospital study found depressive symptoms >6 times more likely (*p* = 0.005) (52). In general, depression and anxiety symptoms should be managed with a combination of psychosocial counseling and antidepressants. Mirtazapine and olanzapine are effective agents for both depression and nausea. A systematic review noted mirtazapine was significantly more likely to cause weight gain or increased appetite in non-cancer patients (53). However, a recent randomized trial showed mirtazapine 15 mg at night did not improve body weight compared to placebo in advanced cancer patients with anorexia-cachexia (54). Despite these mixed findings, mirtazapine is a useful medication because it may improve multiple NIS including depression, anxiety and nausea.

There are no consistently effective therapies for dysgeusia; however, a trial of zinc sulfate is reasonable as this supplement has few side effects in comparison to dronabinol, shown in a small RCT to be beneficial for chemosensory perception (55).

## Conclusion

Nutrition impact symptoms compromise food intake and drive body weight loss. There is increasing evidence that NIS burden is associated with adverse clinical outcomes such as weight loss, poor QoL and even decreased survival. Our case series is limited, but provides a glimpse of the potential for improving NIS and relevant physical performance measures such as SPPB and 6MW. A multimodal interdisciplinary approach (56) to NIS that includes pharmacologic and non-pharmacologic therapies is the ideal, but may be limited by resources, even though assessments are relatively brief and medications are inexpensive. Further research, including clinical trials are necessary to identify the most effective, sustainable approach to management of NIS in daily practice.

## Author Contributions

ED: main conception or design of the work and acquisition, analysis, or interpretation of data for the work. AK and SN: major roles in drafting the work, also in design and revising it critically for important intellectual content. SP: acquisition of data, design and drafting, and revising. All authors provide approval for publication of the content; agree to be accountable for all aspects of the work in ensuring that questions related to the accuracy or integrity of any part of the work are appropriately investigated and resolved.

## Conflict of Interest

The authors declare that the research was conducted in the absence of any commercial or financial relationships that could be construed as a potential conflict of interest.

## Publisher’s Note

All claims expressed in this article are solely those of the authors and do not necessarily represent those of their affiliated organizations, or those of the publisher, the editors and the reviewers. Any product that may be evaluated in this article, or claim that may be made by its manufacturer, is not guaranteed or endorsed by the publisher.
